# Severe Influenza A(H1N1) Virus Infection Complicated by Myositis, Refractory Rhabdomyolysis, and Compartment Syndrome

**DOI:** 10.1155/2019/1540761

**Published:** 2019-01-30

**Authors:** Camila D. Odio, Charisse Mandimika, Thiago A. Jabuonski, Maricar Malinis

**Affiliations:** ^1^Department of Medicine, Yale School of Medicine, New Haven, Connecticut 06510, USA; ^2^Section of Infectious Diseases, Yale School of Medicine, New Haven, Connecticut 06510, USA; ^3^Section of Pulmonary, Critical Care, and Sleep Medicine, Yale School of Medicine, New Haven, Connecticut 06510, USA

## Abstract

Myositis is a rare and morbid complication of influenza infection that can rapidly progress to rhabdomyolysis with acute renal failure. Here, we describe a 35-year-old obese woman with severe influenza A(H1N1) virus infection complicated by myositis, refractory rhabdomyolysis, and compartment syndrome.

## 1. Case

**Figure 1:**
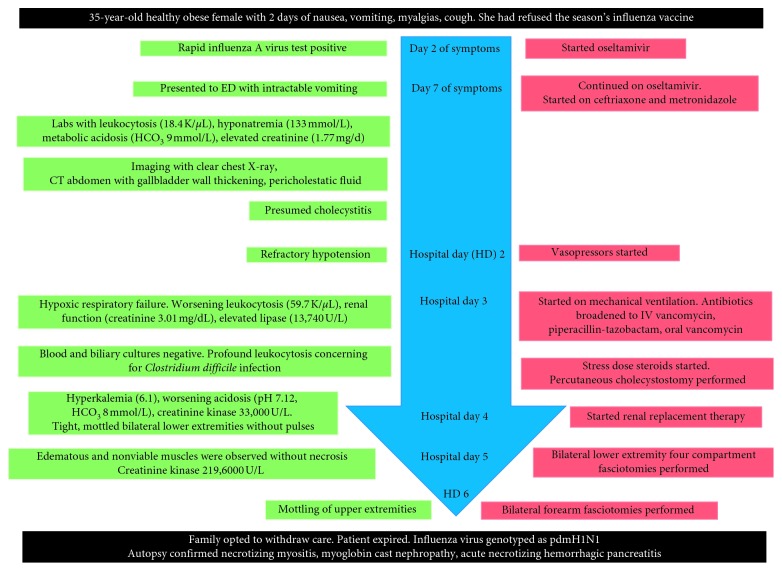
Case timeline.

A 35-year-old obese woman presented to a community clinic in late March with two days of nausea, vomiting, myalgias, and cough and was diagnosed with influenza A virus (Figure 1). She endorsed refusal of the influenza vaccine offered earlier in the season. She was given oseltamivir for a five-day course. On the final treatment day, she presented to the emergency department with inability to tolerate liquids and solids. Laboratory studies showed leukocytosis (18.4 K/*μ*L), hyponatremia (133 mmol/L), metabolic acidosis (HCO_3_ 9 mmol/L), and elevated creatinine (1.77 mg/d) from her baseline of 0.6 mg/dL. Liver function tests, lipase, and creatinine kinase were normal. Human immunodeficiency virus testing was negative. Rapid influenza A virus polymerase chain reaction was positive, and a computed tomography (CT) of the abdomen showed gallbladder wall thickening and pericholestatic fluid. Oseltamivir was continued. Empiric antibiotics, ceftriaxone, and metronidazole were started for presumed cholecystitis.

On hospital day (HD) 2, she developed refractory hypotension requiring vasopressor support. She progressed to hypoxemic respiratory failure on HD 3 and was mechanically ventilated. Laboratory studies revealed worsening leukocytosis (59.7 K/*μ*L with 3.4% bands), worsening renal function (creatinine 3.01 mg/dL), and elevated lipase (13,740 U/L). Antibiotics were broadened to intravenous vancomycin and piperacillin-tazobactam. Due to profound leukocytosis, she was empirically treated for *Clostridium difficile* colitis with oral vancomycin and intravenous metronidazole. Stress dose steroids were started for refractory shock. Blood cultures had no growth. Based on CT imaging suggestive of cholecystitis, percutaneous cholecystostomy was performed. Cultures of biliary fluid were negative.

On HD 4, the patient developed hyperkalemia and worsening acidosis with a pH of 7.12 and HCO_3_ of 8 mmol/L. She was transferred to our institution for emergent renal replacement therapy. Her examination was notable for tight, mottled bilateral lower extremities without pulses and left upper extremity discoloration. To rule out compartment syndrome and myoglobin-associated renal toxicity, creatinine kinase (CK) was sent and this was elevated at 33,000 U/L. On HD 5, CK had progressed to 43,500 U/L. Bilateral lower extremity four compartment fasciotomies were performed at the bedside. Edematous and nonviable muscles were observed without necrosis. Due to nonimprovement, repeat bilateral thigh fasciotomies were performed. Her CK rose to 2196,000 U/L. On HD 6, mottling of upper extremities was noted and bilateral forearm fasciotomies were performed. In light of continued evolving multiorgan failure, her family opted for withdrawal of aggressive interventions, the patient was made comfort measures, and she expired shortly thereafter. Influenza was later genotyped as pdmH1N1. The sample was tested in the Clinical Virology Laboratory at Yale New Haven Hospital, using the seasonal influenza real-time reverse transcriptase (RT) PCR protocol from the Centers for Disease Control and Prevention [1]. Autopsy confirmed necrotizing myositis, myoglobin cast nephropathy, and acute necrotizing hemorrhagic pancreatitis.

## 2. Discussion

The World Health Organization estimates that each influenza season results in 290,000–650,000 deaths globally [2]. Cause of death is often attributable to bacterial coinfection [3]. However, influenza may directly cause mortality through myocarditis, encephalitis, myositis, pancreatitis, and multisystem organ failure [4].

Influenza is responsible for 33–42% of viral myositis complicated by rhabdomyolysis and compartment syndrome [5]. Influenza A virus is more often associated with rhabdomyolysis than influenza B virus [6]. *In vitro*, human muscle [7] and pancreatic [8] cells are susceptible to influenza A virus invasion resulting in cell lysis. *In vivo*, muscle cell death releases myoglobin and osmotically active cellular elements causing edema of the interstitial space within the constrictive compartment. Rising compartment pressure promotes ischemia and tissue necrosis [9]. Our patient developed muscle edema and rising CK despite fasciotomy, suggestive of primary viral myositis.

This severe presentation occurred despite the appropriate initiation of oseltamivir, raising the possibility of viral resistance. The CDC reported that 1% of influenza A(H1N1) viruses sampled during the 2017-2018 season were resistant to oseltamivir, but the majority of these were sensitive to zanamivir [10]. As such, the patient may have benefited from switching to zanamivir, and clinicians managing severe influenza virus infections should consider this option.

Our patient's age, lack of influenza vaccination, and high body mass index (BMI) put her at risk for severe influenza. In 2009, pdmH1N1 had an unusually high attack rate in young people, with 90% of deaths occurring in those younger than 65 years old. This may be due to preexisting immunity in older populations [4]. Obesity is an independent risk factor for influenza-related death [11] and has been associated with hospitalization (6%), ICU stay (11%), and deaths (12%) [12]. The mechanisms underlying this association remain unclear, but studies indicate that adipose interferes with immune signaling and response [13]. Notably, despite influenza vaccination, obese individuals vaccinated are twice more likely to develop infective illness compared to those with normal BMI [14]. However, influenza vaccination does modify disease severity, as vaccinated adults hospitalized for influenza were about 75% less likely to die than unvaccinated patients [15]. Thus, despite vaccine effectiveness of less than 50% [16], patients should be educated regarding the disease modifying benefits.

## 3. Conclusion

Our case highlights rare but deadly complications of influenza infection, including myositis, rhabdomyolysis, compartment syndrome, and necrotizing pancreatitis. These occurred in a young, obese, unvaccinated but otherwise healthy individual despite early institution of antiviral therapy. Influenza vaccine can reduce illness severity, decreasing hospitalizations and saving lives.
